# Does a minimal invasive approach reduce anterior chest wall numbness and postoperative pain in plate fixation of clavicle fractures?

**DOI:** 10.1186/s12891-015-0592-4

**Published:** 2015-05-28

**Authors:** Marc Beirer, Lukas Postl, Moritz Crönlein, Sebastian Siebenlist, Stefan Huber-Wagner, Karl F. Braun, Peter Biberthaler, Chlodwig Kirchhoff

**Affiliations:** Department of Trauma Surgery, Klinikum rechts der Isar, Technical University of Munich, Munich, Germany

**Keywords:** Anterior chest wall numbness, Clavicle fracture, Mini open, Minimal invasive, Locking compression plate

## Abstract

**Background:**

Fractures of the clavicle present very common injuries with a peak of incidence in young active patients. Recently published randomized clinical trials demonstrated an improved functional outcome and a lower rate of nonunions in comparison to non-operative treatment. Anterior chest wall numbness due to injury of the supraclavicular nerve and postoperative pain constitute common surgery related complications in plate fixation of displaced clavicle fractures. We recently developed a technique for mini open plating (MOP) of the clavicle to reduce postoperative numbness and pain. The purpose of this study was to analyze the size of anterior chest wall numbness and the intensity of postoperative pain in MOP in comparison to conventional open plating (COP) of clavicle fractures.

**Methods:**

24 patients (mean age 38.2 ± 14.2 yrs.) with a displaced fracture of the clavicle (Orthopaedic Trauma Association B1.2-C1.2) surgically treated using a locking compression plate (LCP) were enrolled. 12 patients underwent MOP and another 12 patients COP. Anterior chest wall numbness was measured with a transparency grid on the second postoperative day and at the six months follow-up. Postoperative pain was evaluated using the Visual Analog Scale (VAS).

**Results:**

Mean ratio of skin incision length to plate length was 0.61 ± 0.04 in the MOP group and 0.85 ± 0.06 in the COP group (p < 0.05). Mean ratio of the area of anterior chest wall numbness to plate length was postoperative 7.6 ± 5.9 (six months follow-up 4.7 ± 3.9) in the MOP group and 22.1 ± 19.1 (16.9 ± 14.1) in the COP group (p < 0.05). Mean VAS was 2.6 ± 1.4 points in the MOP group and 3.4 ± 1.6 points in the COP group (p = 0.20).

**Conclusions:**

In our study, MOP significantly reduced anterior chest wall numbness in comparison to a conventional open approach postoperative as well as at the six months follow-up. Postoperative pain tended to be lower in the MOP group, however this difference was not statistically significant.

**Trial registration:**

ClinicalTrials.gov NCT02247778. Registered 21 September 2014.

## Background

Fractures of the clavicle account for approximately 3 % of all adult fractures [1], occurring most frequently in young active patients [2] with a male-female ratio of 70:30 [1]. Approximately 80 % of all clavicular fractures are located in the midshaft region. These fractures have traditionally been treated non-operatively. Nowadays randomized clinical trials clearly demonstrated an improved functional outcome and a lower rate of malunion and nonunion following surgical plate fixation in comparison to conservative treatment [3,4]. However, surgical treatment reveals complications such as postoperative infection, nonunion, anterior chest wall numbness [5], incision related postoperative pain and hardware removal [6]. The surgical approach along the long axis of the clavicle anatomically crosses the supraclavicular nerve branches, which provide sensation over the clavicle, the antero-medial shoulder and the anterior chest wall [7]. Although numbness in this region seems not to be associated with a poor clinical outcome [5], an incidence rate of up to 83 % with a mean area of 44 cm^2^ and a high percentage of patients complaining about this issue underline the clinical relevance of this topic [8].

In this context we developed our technique for mini open plating (MOP) of the clavicle to reduce the surgery related soft tissue injury. Therefore the aim of this study was to analyze whether the extent of surgical incision influences the area of anterior chest wall numbness comparing MOP and conventional open plating (COP). Additionally the intensity of postoperative pain in correlation to the length of the skin incision should be observed.

## Methods

### Patients

The inclusion criteria for the study cohort comprised patients with an age between 18 and 75 years having sustained an acute displaced fracture of the clavicle (delay of trauma-repair <14 days), necessitating plate fixation. Written informed consent was obtained from each patient. The individuals shown in the figures gave their written permission to publish their images. All fractures were classified according to the Orthopaedic Trauma Association [9] (OTA). Preoperative standard radiographs of the clavicle (anterior-posterior perpendicular to cassette and anterior-posterior 30 degree angle cephalad) were performed. Patients with a history of any other pathology such as preexisting chest wall numbness, cervical root symptoms, former surgery of the affected shoulder or chest wall, neurological or sensorial deficits or signs of neuropathy were excluded from the study. The study protocol was approved by the local ethics committee (Ethics Committee of the medical faculty, Klinikum rechts der Isar, Technical University of Munich, Germany; study number 5536/12). Between March 2014 and August 2014, 24 displaced fractures of the clavicle in 24 patients (22 men, 2 women) with a mean age of 38.2 ± 14.2 years (22–78 years) were enrolled in the study (see Table 1). Open reduction and internal fixation (ORIF) was performed by using the Synthes® LCP superior anterior clavicle plate in each patient. According to the OTA classification [9] 2 patients had a type B1.2, 5 a type B2.2, 7 a type B2.3, 5 a type B3.1, 1 a type C1.1 and 4 a type C1.2 fracture.

**Table 1:** Patient demographics and outcomes

Group	No	Age	Sex	OTA	Injury mechanism	Incision length (mm)	VAS 2nd pd	Numbness (cm^2^) 2nd pd	Numbness (cm^2^) 6 FU	Plate length (mm)	Incision-plate ratio (mm/mm)	Numbness-plate ratio (mm^2^/mm) 2nd pd	Numbness-plate ratio (mm^2^/mm) 6 FU
**MOP**	1	37	m	B3.1	bicycle accident	70	4.5	6	4	110	0.64	5.45	3.64
	2	23	m	B2.3	bicycle accident	60	2	8	3	94	0.64	8.51	3.19
	3	52	m	B3.1	bicycle accident	75	3	9	4	124	0.60	7.26	3.23
	4	43	m	C1.2	bicycle accident	45	4	0	0	81	0.56	0.00	0.00
	5	28	m	C1.2	football accident	50	2	8	5	81	0.62	9.88	6.17
	6	35	m	C1.2	bicycle accident	45	1	0	0	81	0.56	0.00	0.00
	7	25	m	B3.1	bicycle accident	75	1	8	4	123	0.61	6.50	3.25
	8	25	f	B2.3	fall	70	3	12	10	110	0.64	10.91	9.09
	9	42	m	B2.3	bicycle accident	60	5	10	7	94	0.64	10.64	7.45
	10	46	m	B1.2	bicycle accident	75	2	8	6	110	0.68	7.27	5.45
	11	36	m	B2.3	bicycle accident	65	3	3	2	110	0.59	2.73	1.82
	12	27	m	C1.2	fall	45	1	18	11	81	0.56	22.22	13.58
**Mean**		**35**				**61.3**	**2.6**	**7.5**	**4.7**	**100**	**0.61**	**7.61**	**4.74**
**COP**													
	1	30	m	B1.2	fall	85	2	0	0	94	0.90	0.00	0.00
	2	50	m	B2.3	vehicle accident	115	4	22	19	136	0.85	16.18	13.97
	3	62	m	B2.2	epileptic seizure	115	0	11	10	136	0.85	8.09	7.35
	4	35	m	B2.2	bicycle accident	95	4	0	0	110	0.86	0.00	0.00
	5	28	m	B2.2	football accident	95	6	41	33	110	0.86	37.27	30.00
	6	40	m	B2.2	bicycle accident	105	5	54	39	124	0.85	43.55	31.45
	7	27	m	B2.2	bicycle accident	115	4	73	49	136	0.85	53.68	36.03
	8	78	f	B3.1	fall	90	4	28	24	94	0.96	29.79	25.53
	9	22	m	B2.3	vehicle accident	80	3	7	4	94	0.85	7.45	4.26
	10	62	m	B3.1	motor bike accident	80	4	42	34	110	0.73	38.18	30.91
	11	30	m	C1.1	bicycle accident	55	2	0	0	69	0.80	0.00	0.00
	12	33	m	B2.3	bicycle accident	95	3	34	26	110	0.86	30.91	23.64
**Mean**		**41**				**93.8**	**3.4**	**26**	**19.8**	**110**	**0.85**	**22.1**	**16.93**

*MOP* mini open plating, *COP* conventional open plating, *No* Number, *M* male, *F* female, *OTA* Orthopaedic Trauma Association, *VAS* Visual Analog Scale, *2nd pd* second postoperative day, *6 FU* six months follow-up, Written informed consent was obtained from each patient to publish their data and information

### Surgical technique

All patients underwent ORIF in beachchair position with the affected arm in a mobile position. The decision regarding the surgical approach was based upon the surgeon’s individual preferences, all MOP procedures were done by the senior author (CK). Conventional open plating was performed by two equally experienced upper extremity surgeons (SH, PB). Surgery was done in general anesthesia, perioperative antibiotic prophylaxis was administered either using a 2^nd^ generation cephalosporin or gentamycin. In all patients a standard implant (locking compression plate (LCP), superior anterior clavicle plate, Depuy-Synthes®, 4528 Zuchwil, Switzerland) was used. The duration of the surgical procedure was measured from the time of skin incision until the time of skin suture.

#### Conventional open plating

A transverse skin incision was made along the long axis of the clavicle. The length of the skin incision depended on the estimated plate length according to the fracture pattern. After sharp dissection of the platysma, the soft tissue covering the clavicle was extensively separated from the bone to expose the fracture zone and to prepare the estimated plate position. The fracture hematoma was debrided. To gain anatomical reduction the fracture was temporarily reduced using reduction forceps. The position was checked using fluoroscopy. Lag screws (Depuy-Synthes®, 4528 Zuchwil, Switzerland) were used to fixate wedge fragments. The plate was superiorly centered onto the clavicular shaft and after confirmation of correct plate positioning in fluoroscopy, screw holes were consecutively drilled.

#### Mini open plating

**Fig. 1:**
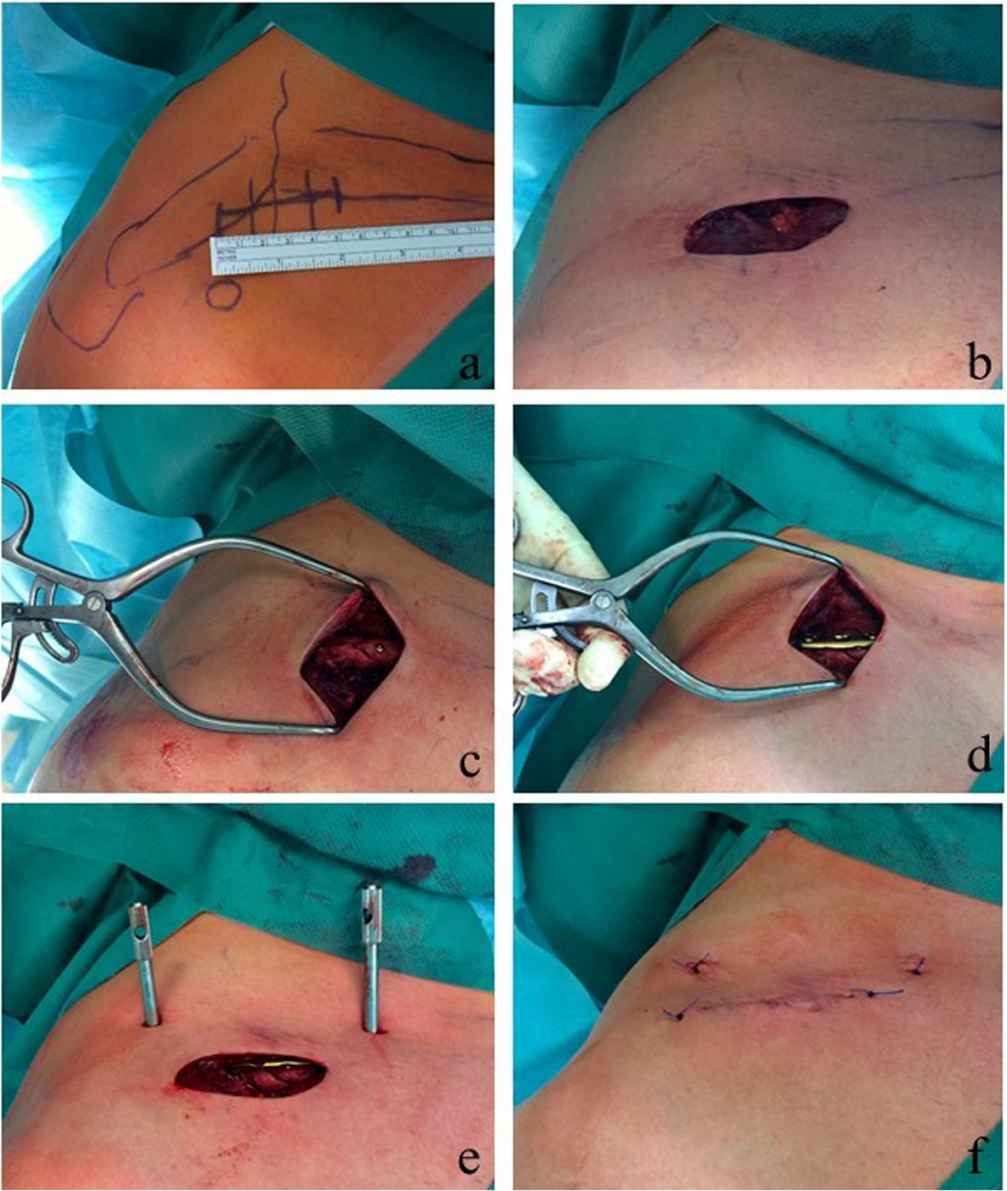
Operation technique in a fracture of the clavicular midshaft (OTA B2.3; patient 11, MOP group)). (**a**) anatomical landmarks and estimated skin incision; (**b**) skin incision to expose the fracture; (**c)** anatomical fixation of the wedge fragments by using two lag screws; (**d)** fixation of the plate; (**e**) stab incisions to drill the medial and lateral plate holes; (**f)** skin suture

In the minimal invasive technique, a small transverse skin incision not extending the length of the fracture zone was made (Fig. 1a + b). After sharp dissection of the platysma and the underlying soft tissue, the fracture was sparingly exposed to debride the fracture hematoma. Anatomical reduction, temporary fixation and fixation of wedge fragments were analogically performed to the COP group (Fig. 1c). The plate was inserted through the small skin incision and superiorly centered onto the clavicular shaft. After confirmation of correct plate positioning in fluoroscopy, screw holes were consecutively drilled. Two additional stab incisions were performed to drill the medial and lateral plate holes (Fig. 1d + e).

### Standardized postoperative protocol

The postoperative analgesia included metamizole and a combination of codeine phosphate and paracetamol. The arm was immobilized in a sling (Medi Sling, Medi SAK, Bayreuth, Germany) and patients started physiotherapy on the first postoperative day following a standard rehabilitation protocol: abduction and flexion were restricted to 90° for the first six weeks. With decreasing pain, this training was progressed with strengthening exercises of the rotator cuff and shoulder muscles. Return to sportive activity of the upper extremities was allowed after another 6 weeks.

### Postoperative assessment

Pain was measured using the Visual Analogue Scale (VAS) on the first, second and fourteenth postoperative day. Values between 0 and 10 could be achieved, whereas *0* stood for *no pain* and *10* stood for *very severe pain*.

**Fig. 2:**
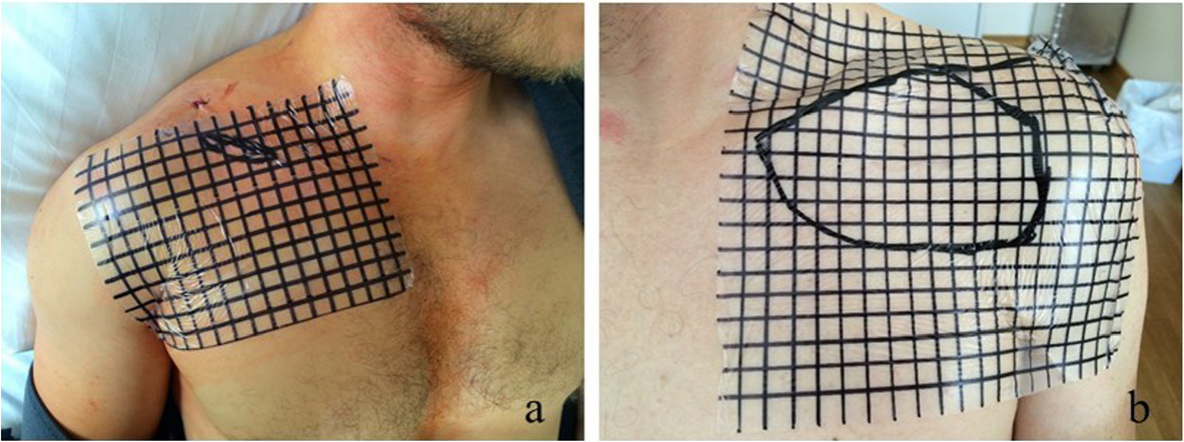
Clinical photograph demonstrating the anterior chest wall numbness on the second postoperative day. (**a**) area of numbness 3 cm^2^ (patient 11, MOP group); (**b**) area of numbness 73 cm^2^ (patient 7, COP group)

Anterior chest wall numbness was assessed on the second postoperative day and six months postoperatively. A grid (1 cm x 1 cm) was superimposed on a transparency slide and temporary put on the patient’s clavicle and anterior chest wall (Fig. 2). The patients were instructed to palpate their chest wall for areas of numbness or decreased sensation to light touch. This line was traced by an examiner onto the transparency slide and measured by summarizing all 1 cm^2^ boxes.

**Fig. 3:**
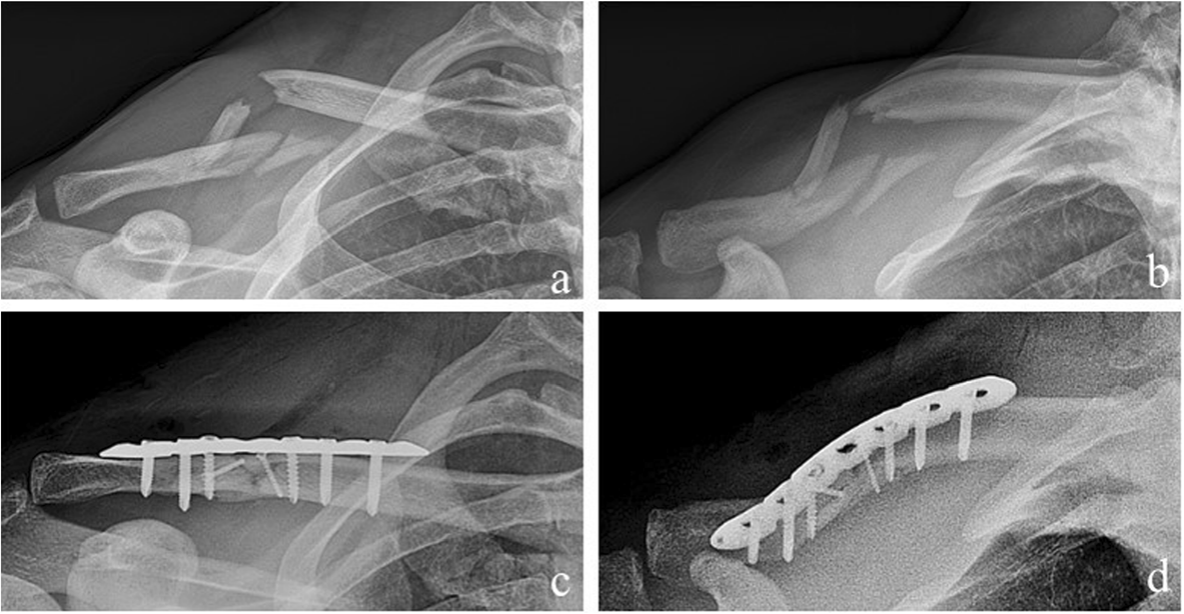
Radiological outcome of a clavicle midshaft fracture OTA B2.3 (patient 11, MOP group). (**a**) + (**b**) preoperative; (**c**) + (**d)** postoperative

### Statistics

We calculated the incision-plate ratio (incision length in mm / plate length in mm) to facilitate the comparability of the skin incision length in both groups. A small ratio implied a small skin incision taking into account the plate length. The numbness-plate ratio (area of numbness in mm^2^ / plate length in mm) was calculated to facilitate the comparability of the area of anterior chest wall numbness in both groups. A small ratio implied a small area of numbness taking into account the plate length. Data are given in terms of the arithmetic mean ± standard deviation. First the data were tested on normality. Data that were not normally distributed were tested with the Mann Whitney U test. Normally distributed data were tested for equality of variances. Data with equal variances were tested with the t-test und data that showed a difference in variances were evaluated with the Welch’s unpaired t-test (two-sample unpooled t-test for unequal variances). The t-test and the Mann Whitney U test were calculated using the software SigmaStat 3.1 (Systat Software Inc., Chicago, USA). The Welch’s t-test was performed with the software QuickCalcs (GraphPad Software, Inc. La Jolla, California, USA). A p-value <0.05 was considered to be significant.

## Results

### Surgery characteristics

The skin incision had an average length of 61.3 ± 12.3 mm in the MOP group and 93.8 ± 17.7 mm in the COP group (p < 0.05). The mean incision-plate ratio in the MOP group was with 0.61 ± 0.04 significantly lower than the mean incision-plate ratio of the COP group which resulted in 0.85 ± 0.06 (p < 0.05 according to the Mann Whitney U test). Surgery had an average duration of 93.5 ± 26.6 minutes in the MOP group and 97.1 ± 24.9 minutes in the COP open group (p = 0.74).

### Postoperative pain and anterior chest wall numbness

The mean VAS was 2.6 ± 1.4 points in the MOP group and 3.4 ± 1.6 points in the COP group on the first postoperative day (p = 0.20; Table 1). On the second and the fourteenth postoperative day there was still no statistical significant difference between the MOP (VAS 1.5 ± 0.5 and 0.8 ± 0.7) and the COP group (VAS 1.8 ± 1.0 and 1.0 ± 0.7) regarding pain. The mean area of anterior chest wall numbness was 7.5 ± 5.0 cm^2^ in the MOP group and 26.0 ± 23.7 cm^2^ in the COP group on the second postoperative day (p < 0.05; Table 1). The mean numbness-plate ratio in the MOP group was with 7.6 ± 5.9 significantly lower than the mean numbness-plate ratio of the COP group which was 22.1 ± 19.1 (p < 0.05 according to the Welsh’s test). At the six months follow-up the mean area of anterior chest wall numbness was 4.7 ± 3.4 cm^2^ in the MOP group and 19.8 ± 17.0 cm^2^ in the COP group (p < 0.05). The mean numbness-plate ratio in the MOP group was with 4.7 ± 3.9 significantly lower than the mean numbness-plate ratio of the COP group which was 16.9 ± 14.1 (p < 0.05).

#### Complications

Figure 3 shows the radiological outcome of a clavicle midshaft fracture OTA B2.3 treated with the minimal invasive technique. There were no major complications such as woundhealing problems, infections, implant failures or revision surgeries to be reported in both groups. Radiologic bony union occurred in all patients after a mean interval of 8–18 weeks postoperative.

## Discussion

In the present study we introduce our recently developed technique for MOP of clavicle fractures. We found a significantly reduced anterior chest wall numbness in comparison to a conventional open approach. Pain showed no clinical or statistical significant difference in the MOP group in comparison to the COP group in the first 14 postoperative days.

Anterior chest wall numbness after plate fixation of clavicular shaft fractures is a very frequent complication in up to 83 % within the first two postoperative weeks. Even after one year 52 % of the patients still report an area of numbness. Although numbness seems not to have an adverse effect on shoulder function, some patients may perceive their degree of numbness as adversely affecting their shoulder function [5]. Another crucial surgery related issue constitutes the postoperative pain caused by the incision related soft tissue trauma.

Fractures of the clavicle mainly occur in young men with a male-female ratio of 70:30 [1]. Although in our study cohort the male gender was predominant, gender was equally distributed in both groups (Table 1). Bike accidents have been the most common trauma mechanism in the presented patient collective. This may result from the increasing popularity of street and mountain bicycling as recreational activities [10].

In general minimal invasive surgery, as presented in our study, offers several advantages such as less tissue dissection, decreased blood loss and potentially less pain which should lead to reduced hospital stays, a quicker recovery and reduced rehabilitation times [11]. However a high technical demand can lead to a long learning curve for the surgeon which can result in prolonged operation times [12]. In our study collective we found no statistical significant differences in comparing the MOP group and the COP group regarding operation time. This finding is similar to the results reported by Kim et al. analyzing a minimal invasive technique in humeral shaft fractures [13].

Another crucial issue in the COP technique is the extensive dissection of the soft tissue to expose the fracture and to prepare the plate position which can lead to further complications. The lacking of an intact soft tissue covering increases the risk of nonunion [14] and infection [15]. Therefore minimal invasive techniques have been developed for further anatomical regions. Pilot et al. [11] compared a conventional posterolateral approach with a minimal invasive anterior approach in hip arthroplasty. However, they didn’t find a benefit of the minimal invasive approach regarding soft tissue damage. These findings were most likely due to the need for a substantial soft tissue traction to become an overview of the anatomical deep position of the hip joint. Due to the subcutaneous position of the clavicle, the surgical approach is not comparable to hip arthroplasty. In our experience, the MOP technique did not require considerable traction of the skin to expose the fracture.

Jung et al. [16] reported a new technique for bridge plating of comminuted shaft fractures of the clavicle to avoid an exposure of the fracture. They stated good functional results after a minimum of 12 months follow-up in a very small patient collective. However, anatomic reduction including anatomically fixation of wedge fragments is highly demanding without exposure of the fracture. Therefore we consider a great risk of healing in malrotation and shortening of the clavicle.

Postoperative pain is caused by the incision related soft tissue damage. Lin et al. [17] stated significant less pain in a minimal invasive technique compared to COP of proximal humerus fractures. In our study postoperative pain was lower in the MOP group but neither clinically nor statistically significant. Even in larger patient collectives a small statistical significant difference in postoperative pain might be clinically not of significance. As an assessment using the VAS is highly subjective [18], these scales are vulnerable to confounders such as comorbidities and data have to be interpreted with caution.

Previous studies have revealed that anterior chest wall numbness from incision related cutaneous nerve damage is a very common complication in plate fixation of clavicle fractures [3,5,8]. The incidence of numbness has been reported to be between 12 and 83 % [5,19]. Even after one year a mean area of 15 cm^2^ is reported in 52 % of the patients [5]. Although postoperative numbness showed no adverse effect on shoulder function measured by validated upper extremity outcome scores [5], some patients reported to be very or extremely bothered by it [8]. In our study collective numbness was significant lower in the MOP group compared to the COP group (p < 0.05). The medial and lateral stab incisions were followed by blunt dissection of the soft tissue down to the periosteum of the clavicle. This technique may increase the chance to protect the branches of the supraclavicular nerve resulting in less anterior chest wall numbness.

### Limitations

First of all the small number of included patients is considered as limitation. The number of patients in the compared groups is relatively low and therefore the reliability of significance is limited. However the literature does not provide any data regarding postoperative numbness and pain in MOP compared to COP of clavicle fractures and therefore we consider our data as relevant. A second limitation concerns the VAS which was used to evaluate postoperative pain. It is a highly subjective measurement tool and the change in the VAS score from pre- to postoperative would be preferable in comparison to a single postoperative development. However, in most trauma units with high patient turnover a scientifically exploitable assessment of preoperative VAS is technically not feasible.

## Conclusions

The surgical treatment of displaced clavicular fractures leads to improved functional results and a lower rate of nonunions in comparison to conservative treatment but it is not without complications. Anterior chest wall numbness and postoperative pain constitute common surgery related complications in plate fixation. Our recently developed MOP technique for displaced clavicle fractures significantly reduced anterior chest wall numbness in comparison to a conventional open approach even at the six months follow-up. Postoperative pain was potentially lower in the MOP group, however this difference was neither clinically nor statistically significant. However, our results still need to be substantiated by analyzing greater patient cohorts which is the focus of our study group.

## Abbrevations

OTA: Orthopaedic Trauma Associaton
LCP: Locking compression plate
mm: millimeter
cm^2^: square centimeter
MOP: Mini open plating
COP: Conventional open plating
yrs: years
